# Prevalencia del consumo de cannabis en Atención Primaria

**DOI:** 10.23938/ASSN.1170

**Published:** 2026-07-24

**Authors:** Alba Sánchez Valverde, Rocío Abarca Dotor, Carlos López Muley, Ximena Soledad Samaniego Vasquez, Neus Parellada Esquius

**Affiliations:** 1 Institut Català de la Salut Gerència d’Atenció Primària i a la Comunitat (GAPiC) Delta EAP Montbaig (Viladecans 3) Viladecans Barcelona España; 2 Institut Català de la Salut Gerència d’Atenció Primària i a la Comunitat (GAPiC) Delta EAP Bartomeu Fabrés i Anglada (Gavà 2) Gavà Barcelona España; 3 Gerència d’Atenció Primària i a la Comunitat (GAPiC) Delta Institut Català de la Salut L’Hospitalet de Llobregat Barcelona España

**Keywords:** Cannabis, Prevalencia, Dependencia a Drogas, Cuestionarios y Encuestas, Atención Primaria, Cannabis, Prevalence, Substance-Related Disorders, Surveys and Questionnaires, Primary Care

## Abstract

**Fundamento:**

Conocer la prevalencia del consumo de cannabis en población adulta, identificar la edad de inicio del consumo y las características sociodemográficas asociadas al mismo, y describir los patrones de riesgo de dependencia.

**Metodología:**

Estudio descriptivo transversal en tres centros de Atención Primaria del área metropolitana de Barcelona. Se seleccionaron personas entre 18 y 45 años mediante muestreo aleatorio simple. Se entrevistaron telefónicamente utilizando las preguntas referentes al consumo de cannabis de ASSIST (*Alcohol, Smoking and Substance Involvement Screening Test*), validada por la Organización Mundial de la Salud. La relación entre las variables sociodemográficas, la prevalencia de consumo y el riesgo de dependencia se analizó mediante regresión logística múltiple.

**Resultados:**

Aceptaron participar 221 (93,3%) de las 237 personas contactadas; el 58,8% eran mujeres, y la media de edad 33,5 años (DT=8,1). El 53,4% refería haber consumido cannabis alguna vez en la vida, con edad de inicio 18,4 años. (DT=4,1); el 25,4% presentaba un riesgo moderado de dependencia según ASSIST. Las probabilidades de consumo fueron superiores en hombres, tras ajustar por edad y nivel económico (OR=2,11; IC95%: 1,21-3,72). El 22,9% había consumido en los últimos tres meses, y la mayoría (81,5%) presentaba riesgo moderado de dependencia. El riesgo moderado de dependencia aumentó en consumidores con estudios de menor nivel.

**Conclusión:**

La prevalencia de consumo de cannabis, superior a las publicadas, fue significativamente mayor en hombres. Una cuarta parte de consumidores presentaban un riesgo moderado de dependencia, mayor a menor nivel de estudios.

## Abstract (en)

**Background:**

To estimate the prevalence of cannabis use among adults attending primary care, identify the age of initiation and associated sociodemographic characteristics, and describe patterns of dependence risk.

**Methods:**

A cross-sectional descriptive study was conducted in three primary healthcare centres in the Barcelona metropolitan area. Adults aged 18 - 45 years were selected by simple random sampling. Telephone interviews used cannabis related questions from the Alcohol, Smoking and Substance Involvement Screening Test (ASSIST), validated by the World Health Organization. Cannabis use prevalence and dependence risk were analysed by sociodemographic characteristics using multiple logistic regression.

**Results:**

Of 237 individuals contacted, 221 participated (93.25%). Among the participants, 58.8% were women, with a mean age of 33.5 years (SD = 8.1). Overall, 53.4% had used cannabis at least once in their lifetime, with a mean age of initiation of 18.4 years (SD = 4.1). Among lifetime users, 25.4% had a moderate risk of dependence. After adjustment for age and socioeconomic status, cannabis use was more likely among men than women (OR = 2.11; 95% CI: 1.21-3.72). Overall, 22.9% had used cannabis within the previous three months, and 81.5% of these users had a moderate risk of dependence. Moderate dependence risk was more frequent with lower educational attainment.

**Conclusions:**

Cannabis use prevalence exceeded that reported in previous studies and was higher in men. One-quarter of lifetime cannabis users had a moderate risk of dependence according to the ASSIST, particularly those with lower educational attainment.

## INTRODUCCIÓN

El cannabis se extrae de la planta *Cannabis sativa* y entre sus principales componentes destacan el tetrahidrocannabinol (THC), responsable de la mayoría de los efectos psicoactivos, y el cannabidiol (CBD), no psicoactivo, compuesto diez veces menos activo que el THC y al que se le atribuyen actividades ansiolíticas y sedantes^1^^,^^2^.

El consumo regular de cannabis se asocia con dependencia, deterioro cognitivo, peor rendimiento académico y laboral, mayor riesgo de trastornos psicóticos, implicación en más conflictos o discusiones y prácticas sexuales de riesgo, así como con policonsumo^3^^-^^6^. Se estima que aproximadamente uno de cada diez consumidores desarrolla un trastorno por consumo de cannabis a lo largo de la vida^6^. Aunque algunos cannabinoides han demostrado utilidad clínica en indicaciones concretas, como control de náuseas y vómitos asociados a quimioterapia, pérdida de apetito en pacientes oncológicos o reducción temporal de la presión intraocular en glaucoma^2^^,^^7^, el consumo no médico continúa representando un importante problema de salud pública^1^^,^^4^.

El cannabis es la droga ilegal más consumida a nivel mundial y la tercera droga más consumida en el mundo, después del tabaco y el alcohol, siendo la sustancia psicoactiva de inicio más precoz, especialmente entre adolescentes y adultos jóvenes. Su consumo ha aumentado en las últimas décadas, tanto con fines recreativos como terapéuticos, acompañado de una disminución de la percepción de riesgo, especialmente entre estudiantes^1^^,^^3^. En 2018, la UNODC (*United Nations Office on Drugs and Crime*) calculó que el 3,9% de la población mundial (192 millones de personas) había consumido cannabis en los últimos 12 meses^1^.

En España, entre 2010 y 2019, la prevalencia de consumo de cannabis durante los últimos 12 meses en estudiantes aumentó tanto en hombres (del 26,8 al 29,2%) como en mujeres (del 23,3 al 25,9%)^8^. El consumo alguna vez en la vida en adultos de 15-64 años ha aumentado del 15 al 40% entre 1995 y 2022, estimándose que el 10-20% de quienes consumen cannabis a diario desarrollan adicción^9^.

En España, el cannabis es la droga ilegal más consumida en todos los grupos etarios. Las encuestas nacionales sitúan la prevalencia de consumo en población adulta (15-64 años), alguna vez en la vida en el 43,7%, el consumo en el último año en el 12,6% y el consumo diario en el 10,5%, manteniendo una tendencia ascendente respecto a ediciones anteriores, con una edad media de inicio cercana a los 18 años y mayor prevalencia en hombres^10^. En estudiantes de 14 a 18 años, los datos del informe ESTUDES 2025 muestran un descenso notable respecto a ediciones previas y mínimos históricos para esta sustancia, con prevalencias del 21,0% alguna vez en la vida, del 15,5% en los últimos 12 meses y del 11,6% en los últimos 30 días, situando la edad media de inicio en 14,8 años^11^.

El abordaje debe ser intersectorial por eso, existen diferentes organismos y comisiones reguladoras que actúan a nivel mundial y europeo en materia de drogas^3^. La Organización Mundial de la Salud (OMS) desarrolló en 1997 la escala *Alcohol, Smoking, and Substance Involvement Screening Test* (ASSIST) para detectar la presencia de consumo de sustancias psicoactivas, diseñada y validada para ser aplicada desde Atención Primaria (AP)^12^^,^^13^, siendo una herramienta diseñada para vincular el cribado a una intervención breve estructurada cuya efectividad ha sido evaluada en ensayos clínicos multicéntricos en AP^14^. Se ha utilizado en un estudio previo en AP en España^15^, y en Canadá se realizó una adaptación, utilizando únicamente el apartado sobre cannabis, detectando una prevalencia de consumo en los últimos tres meses del 8,4%, en mayores de 15 años^16^.

En la consulta de AP, la detección de consumo de alcohol y tabaco está integrada en las actividades preventivas gracias al Programa de Actividades Preventivas y de Promoción de la Salud (PAPPS). Sin embargo, no se dispone de recomendaciones, cuestionarios o registros específicos para el consumo de cannabis, su cribado sistemático no forma parte de las actividades preventivas habituales^17^.

Actualmente, en nuestro país se están implementando proyectos orientados a la prevención, sensibilización, detección precoz de conductas de riesgo e intervención educativa ante sospechas de consumo de drogas en población adolescente. Estas iniciativas se desarrollan principalmente en los centros educativos, mediante la coordinación entre el ámbito escolar y los centros de Atención Primaria (CAP)^18^^,^^19^.

El objetivo de este estudio fue describir la prevalencia y dependencia de consumo de cannabis en población de 18 a 45 años atendida en tres equipos de AP del área metropolitana de Barcelona, y analizar su asociación con las principales características sociodemográficas.

## MATERIAL Y MÉTODOS

Se realizó un estudio transversal entre octubre y diciembre de 2023 sobre consumo de cannabis en personas pertenecientes a tres centros de atención primaria del Instituto Catalán de la Salud del Área Metropolitana de Barcelona: CAP María Bernades en Viladecans, CAP Bartomeu Fabrés Anglada en Gavà, y CAP El Castell en Castelldefels.

La población de estudio se estableció como la población asignada a los tres CAP participantes, con una edad entre 18 y 45 años. Se excluyeron aquellos que manifestaron deseo expreso de no participar o que presentaran dificultades para responder la encuesta.

Dada como referencia una prevalencia de consumo alguna vez en la vida del 40,9%^9^, un error alfa del 5%, una precisión del 7% eran necesarios 190 individuos. Asumiendo un porcentaje de pérdidas de 20%, el tamaño muestral requerido se estimó en 237 personas (Granmo 8.0).

Se llevó a cabo un muestreo aleatorio simple entre los pacientes asignados a los tres CAP. Las personas seleccionadas fueron contactadas telefónicamente por su enfermera asignada, quien les explicó el objetivo del estudio y les invitó a participar. Si aceptaban participar, la enfermera investigadora volvió a contactar nuevamente por teléfono para obtener su consentimiento verbal y, sin tener acceso a su historia clínica, recabar la información. Además, esta enfermera proporcionó a la persona participante el acceso a la web *estudiocribadocannabis.blogspot.com*, donde podría conocer detalles del proyecto, y una dirección de correo electrónico para solventar dudas.

La información se recogió de forma anónima y confidencial.

Las variables en relación al consumo de cannabis reciente y a lo largo de la vida se obtuvieron con la administración de la escala ASSIST, elaborada y validada por la OMS^12^ y validada en castellano^20^. Es un cuestionario de 8 ítems administrado por el profesional para identificar los niveles de consumo reciente y a lo largo de la vida de 10 sustancias (tabaco, alcohol, cannabis, cocaína, anfetaminas u otros estimulantes, ansiolíticos, alucinógenos, inhalantes y otras drogas). En el presente estudio solo se tuvieron en cuenta las siete preguntas sobre el consumo de cannabis (Anexo I), como en el estudio realizado en Canadá por Asbridge y col^16^. Informa de: consumo a lo largo de la vida (sí, no), consumo en los últimos tres meses (sí, no), y -a partir del sumatorio de puntuaciones a las preguntas 2 a 7 - riesgo de dependencia (0 a 3 bajo, 4 a 26 moderado, *≥27* alto), que se dicotomizó en riesgo bajo (≤ 3) y riesgo moderado/alto (>3).

Asimismo, se recogieron otras variables de consumo como:

- Edad de inicio de consumo;
- Modalidad de consumo (inhalada, oral, otras);
- Motivo de inicio de consumo (pregunta abierta que posteriormente se recodificó a terapéutico, social, o no clasificable, otros).

Se recogieron las siguientes variables sociodemográficas mediante un cuestionario *ad hoc*:

- Sexo: hombre, mujer;
- Edad: años cumplidos;
- Nivel económico: bajo, medio, alto (de manera autorreportada y como había sido preguntado en estudios anteriores^15^, que posteriormente se dicotomizó en bajo y medio/alto;
- Nivel de estudios: sin estudios, estudios primarios, secundarios, universitarios, que se categorizó en tres categorías: sin estudios/primarios, secundarios y universitarios;
- Situación laboral: trabajo estable, temporal, desempleo, incapacidad laboral, estudiante, trabajo en casa, jubilación, otros, y no contesta, que se categorizó en tres categorías: activo (estable y temporal), desempleo, otros.

Las variables cuantitativas se describieron mediante media y desviación típica (DT) y las cualitativas con frecuencias absolutas y porcentajes. El análisis del consumo a lo largo de la vida y el grado de dependencia respecto a las variables sociodemográficas se realizó mediante los test de Chi-cuadrado para las variables cualitativas y de U de Mann-Whitney para las cuantitativas, previa comprobación de la falta de normalidad mediante el test de Shapiro-Wilks.

La asociación entre consumo a lo largo de la vida, riesgo de dependencia y variables sociodemográficas de interés se analizó mediante regresión logística múltiple. Las variables independientes incluidas en los modelos se escogieron en base a los resultados del análisis bivariado y según criterios teóricos de ajuste, comparando cada una de las categorías con la categoría de referencia. Se estimaron las *odds ratio* (OR) crudas y ajustadas y sus respectivos intervalos de confianza al 95% (IC95%).

## RESULTADOS

De las 237 personas seleccionadas, 221 (93,25%) respondieron a la entrevista telefónica.

La media de edad fue de 33,5 años (DT=8,1) y el 58,8% fueron mujeres. El 43,5% tenían estudios secundarios y la mayoría declaró un nivel socioeconómico medio (73,8%) y una situación laboral estable (68,3%) (Tabla 1).

**Tabla 1:** Variables sociodemográficas de la muestra

Variables	Total
	n (%)
Edad (años), *media (DT)* (n=221)	33,5 (8,1)
Sexo (mujer), *n (%)* (n=221)	130 (58,8)
Nivel económico, *n (%)* (n=218)	
Alto	7 (3,2)
Medio	163 (73,8)
Bajo	48 (21,7)
Nivel de estudios, *n (%)* (n=214)	
Sin estudios	1 (0,5)
Primarios	31 (14,0)
Secundarios	97 (43,5)
Universitarios	85 (38,5)
Situación laboral, *n (%)* (n=218)	
Trabajo Estable	151 (68,3)
Trabajo Temporal	25 (11,5)
Desempleado	22 (10,1)
Incapacitado	5 (2,3)
Estudiante	14 (6,4)
En casa	1 (0,5)
Activo	176 (79,8)
Desempleo	22 (10,1)
Otros	20 (9,1)

DT: desviación típica.

No hubo diferencias por sexo excepto para la situación laboral (p=0,007), con más mujeres desempleadas (13,8 vs 4,4%) y con trabajos temporales (13,1 vs 8,8%) y menos con trabajo estable (63,8 vs 74,7%) e incapacidad laboral (0,8 vs 4,4%).

Respecto al consumo de cannabis, de los 221 participantes de la encuesta, 118 (53,4%) reportó haber consumido cannabis alguna vez en la vida, siendo el porcentaje de consumidores superior entre hombres que entre mujeres (63,7 vs 46,2; p=0,01). El motivo de inicio de consumo referido fue por curiosidad, social y/o amistades (90%). El consumo de cannabis no se relacionó con la edad, el nivel económico, el nivel de estudios o a la situación laboral (Tabla 2).

**Tabla 2:** Relación de variables sociodemográficas con el consumo de cannabis a lo largo de la vida, global (n=118) y por sexo

Variable	Total	Hombre	Mujer	p
	(n=118)	(n=58)	(n=60)	(*X^2^*)
Edad, *mediana (RIC)*	35	35,5	35	0,63*
	(27,0-40,0]	(26,3-40)	(28,5-40)	
Nivel económico, *n (%)*				0,11
Bajo	27 (56,3)	18 (31,0)	9 (15,0)	
Moderado	89 (52,4)	39 (67,3)	50 (83,3)	
Nivel de estudios, *n (%)*				0,72
Sin estudios o primarios	17 (53,1)	10 (17,2)	7 (11,7)	
Secundarios	55 (56,7)	27 (46,6)	28 (46,7)	
Universitarios	41 (48,2)	18 (31,0)	23 (38,3)	
Situación laboral, *n (%)*				0,14
Activo	98 (83,1)	49 (84,5)	49 (81,7)	
Desempleo	11 (9,3)	3 (5,2)	8 (13,3)	
Otros	9 (7,6)	6 (10,3)	3 (5,0)	

RIC: rango intercuartílico; *: medianas comparadas mediante U de Mann-Whitney.

En el modelo multivariante, solo el sexo retuvo la significación estadística tras ajustar por edad y nivel económico: los hombres presentaron aproximadamente el doble de posibilidades de haber consumido cannabis alguna vez en la vida que las mujeres (OR=2,11; IC95%: 1,21-3,72).

Las características de consumo entre los consumidores se presentan en la Tabla 3. El 22,9% de participantes afirmaba haber consumido cannabis en los últimos tres meses y, aunque no alcanzó significación estadística, la frecuencia en hombres casi duplicó la de mujeres. La edad media de inicio de consumo fue ligeramente superior en mujeres. El consumo fue mayoritariamente por inhalación, seguido del oral, y el consumo en otras formas se consideró negligible (n=1; 0,8%). El porcentaje de personas con consumo de forma oral fue significativamente superior en hombres (31 vs 15%; p=0,03).

**Tabla 3:** Características de los consumidores de cannabis, global y por sexo

Variable	Total	Hombre	Mujer	p
	(n=118)	(n=58)	(n=60)	(*X^2^*)
Consumo en los últimos tres meses	27 (22,9)	17 (29,3)	10 (16,7)	0,102
Edad de inicio, media (DT)*	18,4 (4,1)	18,0 (3,3)	18,8 (5,8)	0,261*
Consumo				
Inhalado	111 (94,1)	56 (96,6)	55 (91,7)	0,267
Oral	27 (22,9%)	18 (31,0%)	9 (15,0%)	0,03

DT: desviación típica; *: comparación mediante U de Mann Whitney.

Entre las 118 personas que habían consumido alguna vez en la vida, el 25,4% presentaba un riesgo moderado de dependencia según la puntuación de la escala ASSIST, sin personas en riesgo alto. Sin embargo, el riesgo moderado de dependencia se triplicó en los consumidores en los últimos tres meses (81,5%). Globalmente, el riesgo de dependencia no se relacionó con otras variables, aunque el porcentaje de consumidores con riesgo moderado fue un 35% mayor en hombres (17 de 58; 29,3%) que en mujeres (13 de 60; 21,7%; p=0,34). La mediana de edad en personas con riesgo moderado fue inferior respecto a aquellas con riesgo bajo, sin alcanzar la significación estadística (32 años; RIC: 27,0-37,8 en comparación a 36 años; RIC: 27-40; U de Mann-Whitney, p=0,058). También se observaron diferencias respecto al nivel de estudios, con un 9,8% (4 de 41) de los universitarios mostrando un riesgo de dependencia moderado, frente al 41,2% (7 de 17) de los que tenían estudios primarios/sin estudios y 32,7% (18 de 55) de los que tenían estudios secundarios (p=0,058). No se encontraron diferencias significativas respecto a la distribución por nivel económico (0,640) o situación laboral (0,640). La relación entre el nivel de riesgo y las variables sociodemográficas en consumidores de cannabis alguna vez en la vida, desglosado por sexo, se muestra en la Tabla 4.

**Tabla 4:** Relación entre variables demográficas y riesgo de dependencia en aquellos que han consumido alguna vez en la vida (n=118), global y por sexo

RIESGO BAJO	Global (n=88)	Hombre (n=41)	Mujer (n=47)	p
				(*X^2^*)
Nivel económico, *n (%)*				0,08
Bajo	21 (23,9)	13 (31,7)	8 (17,0)	
Medio-Alto	63 (71,6)	25 (61,0)	38 (80,9)	
Nivel de estudios, *n (%)*				0,1
Sin estudios o primarios	10 (11,4)	7 (17,1)	3 (6,4)	
Secundarios	37 (42,05)	15 (36,6)	22 (46,8)	
Universitarios	37 (42,05)	17 (41,4)	20 (42,3)	
Situación laboral, *n (%)*				0,21
Activo	75 (85,2)	36 (87,8)	39 (83,0)	
Desempleo	8 (9,1)	2 (4,9)	6 (12,8)	
Otros	5 (5,7)	3 (7,3)	2 (4,3)	
*RIESGO MODERADO*	*Global (n=30)*	*Hombre (n=17)*	*Mujer (n=13)*	(*X^2^*)
Nivel económico, *n (%)*				0,12
Bajo	6 (26,7)	5 (31,2)	1 (0,77)	
Medio-Alto	23 (79,3)	11 (68,8)	12 (92,3)	
Nivel de estudios, *n (%)*				0,24
Sin estudios o primarios	7 (23,3)	3 (17,7)	4 (30,8)	
Secundarios	18 (60,0)	12 (70,6)	6 (46,2)	
Universitarios	4 (13,3)	1 (5,9)	3 (23,1)	
Situación laboral, *n (%)*				0,42
Activo	23 (76,7)	13 (76,5)	10 (76,9)	
Desempleo	3 (10,0)	1 (5,9)	2 (15,4)	
Otros	4 (13,3)	3 (17,6)	1 (7,7)	

En el análisis multivariante, el nivel de estudios mostró significación estadística tras ajustar por sexo y edad. La probabilidad de que el riesgo de dependencia sea moderado respecto a bajo en personas sin estudios universitarios al menos se cuatriplicó tras ajustar por edad y sexo (Tabla 5).

**Tabla 5:** Riesgo de dependencia moderado (respecto a bajo) en función de variables sociodemográficas. Modelos sucesivos de regresión logística

Variables	Modelo 1	Modelo 2	Modelo 3
	OR (IC95%)	OR (IC95%)	OR (IC95%)
Nivel de estudios			
Universitarios	Ref.	Ref.	Ref.
Secundarios	4,50 (1,51-16,72)	4,08 (1,34-15,30)	4,03 (1,32-15,12)
Sin estudios/primarios	6,47(1,63-29,20)	7,14 (1,75-33,21)	6,89 (1,68-32,11)
Edad	-	0,95 (0,89-1,01)	0,95 (0,89-1,01)
Sexo			
Mujer	-	-	Ref.
Hombre	-	-	1,31 (0,53-3,27)

Modelo 1: nivel de estudios; Modelo 2: nivel de estudios + edad; Modelo 3: nivel de estudios + edad + sexo. OR: *odds ratio*; IC95%: intervalo de confianza al 95%. (IC95%).

## DISCUSIÓN

En esta muestra de adultos jóvenes atendidos en AP, más de la mitad de los participantes, un 52,9% de ellos, refirió haber consumido cannabis alguna vez en la vida y, entre estos, el 22,9% afirmó haber consumido en los últimos tres meses (12,2% sobre el total de la muestra). El consumo de cannabis alguna vez en la vida se asoció con el sexo, observándose que la probabilidad de consumir cannabis entre hombres duplicaba la de las mujeres. El riesgo moderado de dependencia, según la puntuación de la escala ASSIST, fue más frecuente en personas con menor nivel de estudios.

Las prevalencias de consumo observadas en este estudio fueron superiores a las descritas en la encuesta nacional EDADES 2024, en población de 15-64 años (43,7% alguna vez en la vida y 10,5% en los últimos 30 días)^10^ y a las estimaciones europeas más recientes para adultos de 15-64 años (donde la prevalencia de consumo alguna vez en la vida es del 31,4% y en el último año es del 8,4% según la *European Drug Report* 2025)^21^. Tal como describe Manthey y col^22^, España presenta prevalencias más elevadas que el resto de Europa y con tendencia creciente en la última década.

Una fuente de variabilidad de resultados entre estudios podría relacionarse con el instrumento de cribado utilizado. El ASSIST ofrece diversas ventajas en el marco de AP, como que fue desarrollado y validado por la OMS en estudios multicéntricos realizados en distintos países^12^^,^^13^, tiene una estructura breve y fácil de aplicar en consulta, y ha mostrado buenas propiedades psicométricas para detectar consumo de sustancias, incluida el cannabis. Además, permite relacionar la puntuación obtenida con una intervención breve protocolizada, cuya eficacia ha sido evaluada en un ensayo clínico multicéntrico aleatorizado en AP^14^. A diferencia de herramientas centradas exclusivamente en cannabis, como el CUDIT-R^23^ o el CAST^24^, el ASSIST evalúa múltiples sustancias en una misma evaluación y hubiera permitido evaluar el policonsumo. Sin embargo, en el presente estudio se optó por preguntar solo por el cannabis ya que, al tratarse de una encuesta telefónica, realizar el ASSIST completo habría incrementado la duración de la entrevista con el consiguiente riesgo de abandono o de no finalización de la encuesta.

Las prevalencias de consumo descritas en el presente estudio fueron superiores respecto a otros estudios también realizados con la herramienta ASSIST. López-Rodríguez y col describieron una prevalencia de consumo de cannabis inferior (5,7%) en pacientes mayores de 18 años en AP en Madrid^15^; esta discrepancia podría explicarse por la antigüedad del estudio y el muestreo por conveniencia entre pacientes que acudían al centro de salud, y también pudo influir que cuando es el propio profesional médico quien realiza las preguntas, se puede ocasionar un sesgo de selección o de falta de verdad por miedo a ser juzgado. Asbridge y col también describieron una prevalencia de consumo del 8,4% en los últimos tres meses en mayores de 15 años en Canadá^16^, inferior a la del presente estudio. Esta podría explicarse por diferencias socioculturales, en los periodos de estudio y en los rangos etarios seleccionados.

La edad media de inicio de consumo en nuestra muestra coincide con la descrita en población adulta española^8^^,^^10^, y es claramente superior a la reportada en estudiantes de secundaria (alrededor de 15 años en ESTUDES 2025^11^ y FRESC^25^) como es esperable.

La modalidad de consumo predominante fue la inhalada, coherente con el patrón mayoritariamente fumado descrito entre los admitidos a tratamiento por cannabis en España (97,5%)^8^.

La prevalencia de consumo alguna vez en la vida fue significativamente superior en hombres, en concordancia con lo descrito en la literatura, señalando desigualdades sociales de género arraigadas en los patrones de conducta sociales^8^^,^^10^^,^^15^^,^^22^. En este estudio no se observó relación con el nivel socioeconómico, aunque se ha descrito una mayor frecuencia de consumo problemático en contextos de mayor vulnerabilidad social^15^^,^^21^. Esta discrepancia podría estar relacionada con las características propias de cada población, con la potencia del tamaño muestral disponible para detectar asociaciones de menor magnitud, y con las limitaciones de las variables sociodemográficas autoinformadas, que inducen un sesgo de mala clasificación no diferencial con tendencia del resultado al nulo^26^.

Respecto a la dependencia, una cuarta parte de quienes habían consumido alguna vez en la vida presentaron un riesgo moderado de dependencia según la puntuación ASSIST. Este hallazgo es relevante, ya que el consumo frecuente se asocia a trastorno por consumo de cannabis, deterioro cognitivo y a un mayor riesgo de trastornos psicóticos^4^^-^^6^^,^^21^^,^^27^. Asimismo, estos resultados se sitúan en el contexto del incremento de las primeras admisiones a tratamiento por cannabis, tanto en España (1.300 en 1996 frente a más de 10.300 en 2019)^8^ como en Europa^21^^,^^22^.

En su revisión, Jan C van Ours y Jenny Williams afirman que la literatura aporta pruebas sólidas de que el consumo temprano de cannabis reduce el nivel educativo alcanzado^28^. El consumo de cannabis puede disminuir la motivación, asociarse a conductas delictivas, y causar problemas de memoria y un bajo rendimiento escolar. El consumo habitual de cannabis en jóvenes se asocia a niveles cognitivos basales más bajos, lo que puede influir en su rendimiento y ponerlos en riesgo de fracaso académico^29^. En el presente estudio, un menor nivel educativo se asoció significativamente con una mayor probabilidad de riesgo moderado de dependencia, sugiriendo que el nivel de estudios podría actuar como un factor sociodemográfico relacionado con la vulnerabilidad al consumo de cannabis^15^^,^^29^^,^^30^.

Este estudio presenta diversas fortalezas. Por un lado, se obtuvo una elevada tasa de respuesta (93,25%), habiendo realizado un muestreo aleatorio simple sobre la población atendida en AP, reduciendo el sesgo de selección habitual en estudios de conveniencia realizados en consulta. Por otro lado, cuantifica el consumo en una franja etaria (18-45 años) que habitualmente es poco caracterizada en las encuestas generales, lo que podría ser relevante al constituir una población diana sobre la que los servicios de AP podrían realizar actividades de detección precoz e intervención. De hecho, hasta donde conocemos, existen escasos estudios realizados en el contexto de la AP española que evalúen específicamente el consumo de cannabis con una herramienta estructurada como el ASSIST^15^.

No obstante, también cabe destacar varias limitaciones. En primer lugar, el diseño transversal impide establecer relaciones causales, a pesar de que las asociaciones encontradas son consistentes con la literatura y presentan una cierta plausibilidad. En segundo lugar, la información fue autorreportada, lo que puede conllevar un posible sesgo de memoria respecto a la edad de inicio, y un sesgo de deseabilidad social que podría haber infraestimado la prevalencia real de consumo. En tercer lugar, por motivos éticos, no se incluyeron menores de 18 años, lo que limita la comparabilidad directa con encuestas escolares. En cuarto lugar, la muestra procede de tres equipos de AP del área metropolitana de Barcelona, por lo que los resultados podrían no ser extrapolables a otros entornos. Y, por último, la utilización de la versión del ASSIST adaptada únicamente a cannabis no permitió valorar el policonsumo -el cual es un fenómeno cada vez más relevante en este perfil de población- ni explorar la potencia del producto consumido, factor de creciente importancia clínica en el contexto europeo actual^21^^,^^22^.

En conclusión, en una muestra de adultos jóvenes atendidos en AP se observó una prevalencia y riesgo de dependencia asociados al consumo de cannabis superiores a los descritos en población general, y una asociación significativa entre un mayor consumo y el sexo masculino, y entre un nivel de dependencia moderada y menor nivel de estudios.

Estos resultados orientan a la necesidad de integrar sistemáticamente una herramienta breve y validada como el ASSIST en las actividades preventivas de AP, y de desarrollar intervenciones breves estructuradas para facilitar la detección precoz y la intervención en este perfil de pacientes. Aunque lo ideal sería abordar todas drogodependencias desde la consulta de AP, debería al menos realizarse intervenciones de detección de las más prevalentes. Como ya hemos indicado, el PAPPS tiene actividades dirigidas a la detección del consumo de alcohol y tabaco, por tanto, debería añadir el consumo de cannabis. Así mismo, desde las instituciones en general y los servicios de AP en particular, se debe continuar trabajando conjuntamente en la prevención del consumo de cannabis, informando de forma veraz a la población diana susceptible -es decir, los adolescentes-, fomentando hábitos de vida saludables y promoviendo la psicoeducación para que conozcan los riesgos y consecuencias de su consumo.

## Data Availability

Se encuentran disponibles bajo petición a la autora de correspondencia.

## ANEXO I

### Test ASSIST: Apartado cannabis

**Table:**

Preguntas	Respuesta	Puntuación
Pregunta 1. ¿A lo largo de la vida ha consumido cannabis?	No	0
	Sí	3
Si contesta NO, se finaliza la entrevista. Agradeciendo su colaboración.		
Si contesta SÍ, se sigue con la entrevista.		
Pregunta 2. En los últimos 3 meses, ¿con qué frecuencia ha consumido cannabis?	Nunca	0
	Una o dos veces	2
	Mensualmente	3
	Semanalmente	4
	A diario o casi todos los días	6
Si contesta NUNCA, pasar directamente a la pregunta 6.		
Si contesta cualquiera de las otras respuestas, continuar con pregunta 3.		
Pregunta 3. En los últimos 3 meses, ¿con qué frecuencia ha sentido un fuerte deseo o ansia de consumir cannabis (marihuana, mota, hierba, hachís	Nunca	0
	Una o dos veces	2
	Mensualmente	3
	Semanalmente	4
	A diario o casi todos los días	6
Pregunta 4. En los últimos 3 meses, ¿con qué frecuencia el consumo de cannabis le ha causado problemas de salud, sociales, legales o económicos?	Nunca	0
	Una o dos veces	4
	Mensualmente	5
	Semanalmente	6
	A diario o casi todos los días	7
Pregunta 5. En los últimos 3 meses, ¿con qué frecuencia dejó de hacer lo que habitualmente se esperaba de usted por el consumo de cannabis?	Nunca	0
	Una o dos veces	5
	Mensualmente	6
	Semanalmente	7
	A diario o casi todos los días	8
Pregunta 6. ¿Un amigo, un familiar o alguien más alguna vez ha mostrado preocupación por sus hábitos de consumo de cannabis?	No, nunca	0
	Sí, en los pasados 3 meses	6
	Sí, pero no en los pasados 3 meses.	3
Pregunta 7. ¿Ha intentado alguna vez reducir o eliminar el consumo de cannabis y no lo ha logrado?	No, nunca	0
	Sí, en los pasados 3 meses	6
	Sí, pero no en los pasados 3 meses.	3
Puntuación total ASSIST de las preguntas 2-7	0-3	Bajo riesgo/no intervención
	4-26	Riesgo moderado/intervención breve
	≥27	Alto riesgo/intervención intensiva
